# Carotenoids: potential allies of cardiovascular health?

**DOI:** 10.3402/fnr.v59.26762

**Published:** 2015-02-06

**Authors:** Maria Alessandra Gammone, Graziano Riccioni, Nicolantonio D'Orazio

**Affiliations:** 1 Human and Clinical Nutrition Unit, Department of Medical, Oral and Biotechnological Sciences, University G. D'Annunzio, Chieti, Italy; 2 Cardiology Unit, Cardiology Department, San Camillo De Lellis Hospital, Manfredonia, Italy

**Keywords:** carotenoids, cardiovascular, lycopene astaxanthin, zeaxanthin, lutein, fucoxanthin, beta-cryptoxanthin, lycopene

## Abstract

Carotenoids are a class of natural, fat-soluble pigments found principally in plants. They have potential antioxidant biological properties because of their chemical structure and interaction with biological membranes. Epidemiologic studies supported the hypothesis that antioxidants could be used as an inexpensive means of both primary and secondary cardiovascular disease (CVD) prevention. In fact, the oxidation of low-density lipoproteins (LDL) in the vessels plays a key role in the development of atherosclerotic lesions. The resistance of LDL to oxidation is increased by high dietary antioxidant intake, so that carotenoids, as part of food patterns such as the Mediterranean diet, may have beneficial effects on cardiovascular health too. Further properties of carotenoids leading to a potential reduction of cardiovascular risk are represented by lowering of blood pressure, reduction of pro-inflammatory cytokines and markers of inflammation (such as C-reactive protein), and improvement of insulin sensitivity in muscle, liver, and adipose tissues. In addition, recent nutrigenomics studies have focused on the exceptional ability of carotenoids in modulating the expression of specific genes involved in cell metabolism. The aim of this review is to focus attention to this effect of some carotenoids to prevent CVD.

Carotenoids are largely widespread in the vegetable kingdom and are found in high concentrations in algae and microorganisms. Humans and other animals cannot synthesize them, so they are necessary in their diet. The great family of carotenoids encompasses more than 500 members, about 50 are present in our food, but only 20 are absorbed in the intestine and reach our body tissues (1). Carotenoids are classified, according to their chemical structure, into carotenes and xanthophylls. Carotenes include beta-carotene and lycopene and xanthophylls include lutein, fucoxanthin, canthaxanthin, zeaxanthin, beta-criptoxanthin, capsorubin, and astaxanthin (2, 3). The antioxidant properties of carotenoids have been considered the main mechanism of their beneficial health effects (4). However, it would be difficult to explain all the physiological effects of carotenoids merely by their antioxidant activity. Carotenoid serum concentration reflects diet, since we only get carotenoids from food, with the exception of dietary supplements. However, carotenoids are part of a complex metabolism and respond to systemic forces. Circulating carotenoids will in particular be lower if they exist in a highly free radical (FR) environment. In particular, they are lower in cigarette smokers, and even in passive smokers, than in non-smokers, while correct dietetic patterns, such as ‘Mediterranean diet’, are more closely related with circulating carotenoids, compared to the total carotenoid content of the eaten food. Carotenoids have been found in various human organs and tissues, such as human liver, lung, breast, cervix, skin, adipose, and ocular tissues. The major storage places are represented by adipose tissue and liver. Tissues containing large amounts of low-density lipoprotein (LDL) receptors seem to accumulate high levels of carotenoids, probably as a result of non-specific uptake by lipoprotein carriers. Nutrition plays a significant role in the prevention of many chronic diseases such as cardiovascular disease (CVD), especially coronary heart disease (CHD) and stroke (5). In particular, thrombotic disease is the consequence of traditional risk factors such as smoking, hypertension, hyperlipidemia, insulin resistance, diabetes, and obesity. Recently, novel risk factors were investigated such as high sensitive C-reactive protein (hs-CRP) and other markers of inflammation, such as homocysteine, and lipoprotein-a (6). Along with genetic factors and age, lifestyle and diet are also considered important risk factors (7). Dietary interventions should be the initial step in the treatment of CVD. In particular, carotenoids, a group of phytochemical substances that are responsible for the color of some foods, may play an important role in the prevention of human diseases and in the maintenance of good health (8): as part of a balanced diet, these nutrients are responsible, in part and through a synergistic cooperation with other useful antioxidants, for the benefit of some food patterns such as the Mediterranean diet.

## Astaxanthin

Astaxanthin is a red soluble pigment belonging to the family of xanthophylls, abundant in the marine world, where it can be found in microalgae, plankton, krill, fish, and other seafood. It is responsible for the typical coloration of salmon and crustaceans (9). Humans are not able to synthesize astaxanthin and need to take it from food. Once introduced, the duodenum absorbs it, and it passes into the blood, reaches the liver, where it binds to lipoproteins before being distributed throughout the whole body. Through its high lipophilicity, it can also cross the blood–brain barrier and reach the brain and eye structures. Astaxanthin cannot be converted to vitamin A, which means that excess intake will not cause hypervitaminosis A toxicity (9, 10). In 1987, the United States Food and Drug Administration approved astaxanthin as a feed additive for use in the aquaculture industry and in 1999 it was approved as a nutraceutical human dietary supplement. The microalgae *Haematococcus pluvialis*, one of the most important species for its production, is a freshwater species of Chlorophyta that produces the astaxanthin isomer (3*S*, 3*S*′), which is the form found in wild salmon. Astaxanthin used in nutritional supplements is usually a mixture of configurational isomers produced by *Haematococcus pluvialis* (11). Astaxanthin showed potential capacity for protecting the organism against a wide range of diseases, and considerable promising applications in the prevention and treatment of various oxidative stress-related diseases, such as cancers, chronic inflammatory diseases, metabolic syndrome, diabetes, diabetic nephropathy, liver and gastrointestinal diseases, neurodegenerative diseases, and even CVD (12–15), considering that oxidative stress is a pathophysiological process involved in atherosclerotic vascular damage (16). Astaxanthin has a strong antioxidant activity, is a great FRs scavenger and in particular a potent quencher of radical oxygen species (ROS) and nitrogen oxygen species (NOS) (17). Several studies showed that its unique chemical structure makes it more stable within the cell membranes, thus allowing a more efficient antioxidant action. Intracellular FRs are captured by astaxanthin and transferred to the extracellular side where they are inactivated by the action of water-soluble antioxidants such as vitamin C. In this way, it is possible to explain the close synergy between hydrosoluble and liposoluble antioxidants (18). Many works highlight that astaxanthin improves blood lipid profile by reducing LDL-cholesterol (LDL-C) and triglycerides (TG), increasing high-density lipoprotein cholesterol (HDL-C), and decreasing markers of lipid peroxidation (19), inflammation (20, 21), and thrombosis (22). Yoshida et al. (23) demonstrated in a randomized placebo-controlled human study (61 non-obese subjects aged 20–65) that astaxanthin consumption (0, 6, 12, and 18 mg/day for 12 weeks) ameliorates TG and HDL-C in correlation with increased adiponectin in humans. Iwamoto et al. (19) demonstrated a significant inhibition of LDL-C oxidation in 24 healthy volunteers who took doses of astaxanthin (from 1.8 to 21.6 mg/day for 2 weeks). Park et al. (24) studied the effects of dietary supplementation of astaxanthin (0, 2, and 8 mg/day, over 8 weeks) on oxidative stress and inflammation: participants taking 2 mg/day had lower hs-CRP at Week 8: the hs-CRP is considered an important indicator of heart disease. There was also a decrease in DNA damage measured using plasma 8-hydroxy-2′-deoxyguanosine after 4 weeks’ treatment. Another potential benefit to cardiovascular health is the fact that astaxanthin lowers the blood pressure and the risk of heart attack for its modulatory effects on nitric oxide (NO) (25, 26); in fact, Hussein et al. (27) found that oral administration of astaxanthin for 14 days significantly lowered the arterial blood pressure in spontaneously hypertensive rats, and the long-term administration for 5 weeks could also delay the incidence of stroke in spontaneously hypertensive rats (27). Also, Pashow et al. suggested that there might be a potential therapeutic role for astaxanthin in the management of myocardial injury, oxidized LDL, and re-thrombosis after thrombolysis, as well as other cardiac diseases, such as atrial fibrillation (17). However, these short-term benefits *in vitro* and in animal models are not sufficient to affirm undoubtedly that carotenoids are clearly beneficial for CVD and other diseases, in particular, if we consider that their supplemental, isolated form in doses much larger than usual in diet have not frequently showed long-term benefits (28) against several null or adverse studies of some carotenoids supplements (29–31).

## Fucoxanthin

Fucoxanthin is an orange carotenoid present in edible brown seaweeds, such as *Undaria pinnatifida (Wakame)*, *Hijikia fusiformis (Hijiki), Laminaria japonica (Ma-Kombu)*, and *Sargassum fulvellum*. It belongs to the class of non-pro-vitamin A carotenoids, and is a xanthophylls, whose distinct structure includes an unusual allenic bond, epoxide group, and conjugated carbonyl group in polyene chain with antioxidant properties (32, 33). Dietary administrated fucoxanthin is converted to amarouciaxanthin A via fucoxanthinol in mice (34, 35). This metabolic conversion, requiring NAD(P)+ as cofactor, was also observed in human hepatoma cell (HepG2) (36). Dietary fucoxanthin is hydrolyzed to fucoxanthinol in the gastrointestinal tract by digestive enzymes such as lipase and cholesterol esterase and then converted to amarouciaxanthin A in the liver (37). Thus, these metabolites are considered to be the active forms that exert physiological functions in the body. Amarouciaxanthin A is stored in abdominal white adipose tissue (WAT), fucoxanthinol in other tissues (38). Currently, there are few data about pharmacokinetics of fucoxanthin and its metabolites in human subjects. Recent studies reported that fucoxanthinol was detectable in human plasma after daily intake of Wakame. Data about pharmacokinetics of fucoxanthin demonstrated that bioavailability and metabolism of fucoxanthinol is higher in humans than in mice (39–41), but fucoxanthin absorption rate is generally affected by the composition of food matrix: for example, its solubility in soybean oil and in other vegetable oils is very low, while fucoxanthin can easily dissolve in medium-chain triacylglycerols (MCT) or in fish oil (42). Fucoxanthin acts on the reduction of major cardiovascular risk factors, such as obesity, diabetes, high blood pressure, chronic inflammation, plasma and hepatic triglyceride, and cholesterol concentrations (43–45). The identification of substances that can decrease or prevent obesity remains the main goal of medical research. Adaptive thermogenesis by uncoupling protein-1 (UCP1) could be a physiological defense against obesity (43). UCP1 expression is known to be a significant component of whole body energy expenditure, and its dysfunction contributes to the development of obesity (46). In fact, during normal metabolism, the body produces heat, a process also called thermogenesis, and fucoxanthin increases the amount of energy released as heat in fat tissue. UCP1 induction by fucoxanthin metabolites accumulated in WAT is of great interest because UCP1 is normally expressed only in brown adipose tissue (BAT) and not in WAT. This protein, situated in the mitochondrial inner membrane, dissipates the pH-gradient generated by oxidative phosphorylation, releasing chemical energy as heat. UCP1 expression in WAT by fucoxanthin intake leads to oxidation of fatty acids and heat production in WAT (47). Fucoxanthin was found to induce both protein and mRNA expression of UCP1 in WAT (44). This finding will give a clue for new dietary anti-obesity therapies. All these promising scientific findings have been obtained through animal studies, and therefore the fucoxanthin, to keep its promises of anti-obesity nutraceutical, needs to be extensively tested on humans. Only one study has been conducted in humans, which has evaluated the effectiveness of fucoxanthin supplementation for weight loss. In this study, Abidov et al. (48) tested the fucoxanthin in 151 non-diabetic, obese premenopausal women. Three quarters of participants were affected by non-alcoholic fatty liver disease (NAFLD), while the remaining had a normal liver function. The women were divided into two groups and invited to take respectively 600 mg of Xanthigen, which contains 300 mg pomegranate seed oil (PSO) and 300 mg brown seaweed extract containing 2.4 mg fucoxanthin or a placebo for 16 weeks. The diet was reduced to 1,800 kcal per day and was composed of 50% carbohydrates, 30% protein, and 20% fat. The results provided a significant reduction of body weight, fat, and systolic/diastolic blood pressure; decreased levels of TG and of some enzymes (CRP, glutamic pyruvic transaminase (GPT), glutamic oxaloacetic transaminase (GOT), gamma-glutamyl transpeptidase (gamma-GT)), and significant increase in resting energy expenditure (REE) measured by indirect calorimetry. The 16-week supplementation with 4.0 mg/day fucoxanthin showed an important increase in REE and an even greater increase in the group taking fucoxanthin at a dose of 8 mg. Obese patients with NAFLD commonly present elevated markers of liver inflammation and injury, including CRP, GOT, GPT, and gamma-GT (49). A significant reduction in body weight and fat in obese individuals results in the downregulation of inflammatory markers and prevent metabolic syndrome. It has been demonstrated that increased GPT and CRP plasma levels are associated with decreased hepatic insulin sensitivity, insulin resistance, and an increased risk for the onset of metabolic syndrome and type 2 diabetes. The potential antidiabetic effects of fucoxanthin are attributable to the ability of this molecule to induce weight loss and WAT reduction. The adipocyte has recently been recognized as an endocrine cell for its role in the secretion of biologically active mediators, termed adipokines/chemokines, including leptin, adiponectin, resistin, tumor necrosis factor-alpha (TNF-alpha), and monocyte chemoattractant protein-1 (MCP-1). Some adipokines are reported to alter insulin sensitivity and glucose and lipid metabolism in muscle, liver, and adipose tissues (50). The participation of macrophages in inflammatory responses by the release of pro-inflammatory mediators (TNF-alpha and MCP-1) under obesity conditions has also been reported. The chronic low-grade inflammation elicited by pro-inflammatory mediators in the WAT leads to insulin resistance (51). A recent study, using cultivated cells, showed that fucoxanthinol prevents inflammation and insulin resistance also by inhibiting NO and PGE2 production through the downregulation of inducible nitric oxide synthase (iNOS) and COX-2 mRNA expression as well as adipocytokine production in WAT. iNOS is an enzyme that produces NO, which is a FR molecule related to the pathogenesis of inflammation. The overexpression of iNOS mRNA has been observed in WAT of obese mice and adipocytes (52). An interesting, extra metabolic benefit of fucoxanthin administration in rodents is the promotion of the synthesis of docosahexaenoic acid (DHA) in the liver, resulting in improvements in lipid profile (53). Experiments on stroke-prone spontaneously hypertensive rats (SHRSP) show the possible protective role of fucoxanthin in CVD. Thirty-three male SHRSP rats, 5 weeks of age, were divided into three groups: 1) kaolin group, which was given a normal diet (kaolin is a non-nutrient material); 2) Wakame (*Undaria Pinnatifida*) group (normal diet containing Wakame powder); and 3) cellulose group (normal diet containing cellulose). In this study, Wakame delayed the incidence of stroke signs and increased the life span of SHRSP (54). Clinical research also indicated that the metabolic boost from taking fucoxanthin did not stimulate the central nervous system, meaning it did not cause jitters or loss of sleep such as caffeine, nicotine, or thyroid hormones. So the fucoxanthin may have a potential role in modulation and prevention of human diseases, particularly in reducing the incidence of CVD (55). As a carotenoid, fucoxanthin is a powerful antioxidant that protects cells from FRs damage. A diet rich in fucoxanthin could help to reduce body fat accumulation and to modulate blood glucose and insulin levels, through the regulation of cytokine secretions from WAT. Fucoxanthin proved safe with no side effects, and even provided other health benefits, including improved cardiovascular health, reduction of inflammation (a major cause of heart disease), healthy cholesterol and TG levels, improvements in blood pressure levels, and healthy liver function (56).

## Lycopene

Lycopene is the pigment responsible for the red color in some fruits and vegetables, which can be found in high concentration in tomato products, red grapefruits, and watermelons (57–60). It is an unsaturated carotenoid, resulting in an efficient antioxidant, and consumption can prevent both aging and CVD (61–65) because of its important bioactivities. It seems to eliminate ROS, to inhibit lipid peroxidation, and even to reinforce the immune system (57–60). In fact, it is a lipophilic molecule transported in blood by lipoproteins which accumulates in human tissues, also in the vasculature (66). Low plasmatic levels of antioxidant vitamins A, E, beta-carotene, and lycopene were shown to be associated with early carotid atherosclerotic lesions (67). In particular, the Rotterdam Study reported that lycopene was inversely associated with the calcified plaques of the abdominal aorta (62), and several works have clearly associated lycopene with reduced carotid intima-media thickness and lower incidences of cardiovascular accidents, such as CHD and stroke (63). The antiatherogenic effect of lycopene is associated with anti-inflammatory activities, better lipid homeostasis (65) (determining higher serum HDL-C, lower ratio of total cholesterol to HDL, and lower triacylglycerols), antioxidation and consequent inhibition of LDL peroxidation, and protection of vascular endothelium. In fact, lycopene was shown to decrease vascular oxidative stress and inflammation, blood lipid biomarkers of oxidative stress *in vivo* (68), and attenuate adhesion molecule expression and interactions between monocytes and endothelial cells (69). This anti-inflammatory effect was realized by inhibiting IL-1 secretion, which is a key factor in inflammatory processes inducing the synthesis of other pro-inflammatory cytokines, adhesion molecules, chemotactic factors, and acute-phase proteins (70, 71). The production of flogistic mediators (such as IL-1, IL-6, and TNF) and the following recruitment of leukocytes to the intima is involved in the early formation of atherosclerotic lesions, conducing to the chronic inflammatory process of atherosclerosis (72). In addition, lycopene displayed positive effects on the maintenance of NO levels, contributing to vasodilatation, even resulting in a more effective slowing of the progression of atherosclerosis than by fluvastatin, thereby reducing the cardiovascular risk (73). These results suggest the beneficial effect of higher serum and tissue levels of lycopene: for this reason dietary intake of lycopene (especially if diet is also rich in extra-virgin olive oil) (74) or lycopene supplementation (75) seems to decrease the risk of CVD (62, 76). However, several factors can affect lycopene bioavailability and absorption: season, dietary sources, food composition, and processing such as cooking or heating (77), which were reported to transform all-*trans*-lycopene to *cis*-lycopene (78), which is better absorbed. So higher serum levels of lycopene were found when tomatoes have been consumed cooked rather than raw (79). On the contrary, too much prolonged heat treatment (more than 2 h at 100°C) of tomatoes decreases the total carotenoid content, also affecting the beneficial effects against dyslipidemia and cardiovascular risk (80).

## Lutein

Lutein is a pigment (xanthophyll) and a dietary oxygenated carotenoid consisting of 40-carbon hydroxylated compounds found in the human retina in high concentration (81). It is an isomer of the carotenoid zeaxanthin, with identical chemical formulas. Similarly to zeaxanthin, it can just be obtained from supplements or diet, found in several foods, such as yellow corn, egg yolk, orange juice, honeydew melon, and other fruits (82), but especially occurring in dark green vegetables such as turnip greens, kale, parsley, spinach, and broccoli (83). Lutein, which has been shown to prevent lipid peroxidation (84), is well-known to be protective against age-related macular degeneration (AMD) and senile cataract (85, 86), whose major risk factor is oxidative stress (87). In fact, lutein has a strong ROS scavenger capacity (88, 89), blocks the activation of the ubiquitous nuclear transcription factor NF-kB playing a key role in many pathological reactions (90) and the degradation of the inhibitor kB (I-kB) (91). When I-kB is dissociated from the NF-kB complex by lutein, NF-kB can translocate into the nucleus, decreasing inducible gene transcription and synthesis of inflammatory markers such as cytokines, chemokines, and iNOS (92). The final effect of lutein involves not only decreasing the concentrations of TNF-alpha, interleukin 6 (IL-6), prostaglandin 2 (PGE-2), monocyte chemotactic protein 1 (MCP-1), and macrophage inflammatory protein 2 (MIP-2) (91) but also reducing oxidative stress. However, its antioxidant and anti-inflammatory capacity have been shown to have a positive influence not only on eyes but also in promoting cardiovascular health and decreasing the risk of CAD (93). Recent studies showed that plasmatic lutein and oxidized LDL were inversely correlated, suggesting its potent antioxidant and anti-inflammatory effects also on aortic tissue, which may protect against development of atherosclerosis (94). In fact, several works suggest that in atherosclerosis, serum levels of lutein were significantly lower than that in controls, and that it was inversely associated with carotid stiffness (95). In addition, the ARIC (96) and the CUDAS studies (97) displayed a possible beneficial effect of a lutein-rich diet, and also the Los Angeles Atherosclerosis Study showed the inverse association between plasmatic lutein and atherosclerosis, so that higher levels of lutein (such as zeaxanthin and beta-carotene) may be protective against early atherosclerosis (98, 99). A beneficial effect of lutein on heart and blood vessels was also related to prevention of hypertension. A higher concentration of this carotenoid was generally inversely associated with an increase in systolic blood pressure and incidental hypertension. Subjects with higher lutein levels seem to show lower baseline blood pressure, generally with lower risk for future hypertension, independent of smoking status (100). In addition, lutein seems to exert a cardioprotective effect, bringing therapeutic benefit in the treatment of cardiovascular complications. In fact, FRS and oxidative stress are known to be mediators of myocardial ischemia/reperfusion damage (the restoration of blood flow to ischemic regions, with increased generation of highly reactive oxygen species) (101–103). Lutein protects myocardium from ischemia/reperfusion injury by decreasing oxidative stress and myocytes apoptosis (104). Limiting myocardial injury may prevent contractile dysfunction, reducing morbidity and mortality associated with CAD (105).

## Zeaxanthin

Like lutein, zeaxanthin is an oxygenated non-pro-vitamin A carotenoid that consists of a 40-carbon hydroxylated compound (106). Major dietary sources of this xanthophyll in the diet include corn, eggs, orange juice, honeydew melon, and dark green leafy vegetables such as kale, turnip greens, spinach, and broccoli (83). The area of the retina serving central vision is known as the macula lutea because of its yellow coloration from lutein; however, it also contains zeaxanthin. The relative concentration of lutein to zeaxanthin in the macula is distinctive: zeaxanthin is more centralized and lutein predominates toward the outer area of the macula. A xanthophyll-binding protein may explain the differences among people to accumulate these carotenoids into eye tissues. Increased lutein and zeaxanthin intake from both food sources and supplements is positively correlated with increased macular pigment density, which is theorized to lower risk for macular degeneration; in fact, several population studies suggest lower rates of AMD among people with higher levels of zeaxanthin in diet and blood. Possible mechanisms of action for these carotenoids include antioxidant protection of the retinal tissue and the macular pigment filtering of damaging blue light (107). In addition to quenching reactive oxygen species directly, zeaxanthin may prevent protein, lipid, or DNA from oxidative damage by regulating other cellular antioxidant systems. Glutathione is one of the major intracellular antioxidants not only in the lens and plays an important role in protecting cells from oxidative damage (108). In this sense, the protective effects of zeaxanthin, against protein oxidation, lipid peroxidation, and DNA damage resulted to be comparable to α-tocopherol: supplementation with zeaxanthin or α-tocopherol decreases oxidized glutathione (GSSG) and increases the intracellular reduced glutathione (GSH) levels and GSH/GSSG ratio, especially in response to oxidative stress. Thus, zeaxanthin acts as an antioxidant in a directly or indirectly, by regulating glutathione synthesis and therefore glutathione levels. As a consequence, intracellular redox status upon oxidative stress improves and the susceptibility to H_2_O_2_-induced cell death reduces (82).

Zeaxanthin is not only implied in the health of the eye but also in cardiovascular aspects, such as beta-carotene; zeaxanthin, which resulted inversely correlated with right common carotid artery stiffness; pulse wave velocity; and elastic modulus. The Beijing atherosclerosis study and the Los Angeles Atherosclerosis study also found the inverse association between plasma lutein and early atherosclerosis, and their further studies showed that higher levels of plasma zeaxanthin may be protective against early atherosclerosis (99). These results indicated that zeaxanthin might be beneficial to arterial health.

## Beta-cryptoxanthin

Beta-cryptoxanthin is a xanthophylls and one of the lesser-known carotenoids, whose best food sources are oranges, peach, tangerines, and tropical fruits such as papaya. It also has pro-vitamin A activity and seems to have protective health action. Many epidemiological studies showed that dietary beta-cryptoxanthin is associated with improved respiratory function and lower rates of lung cancer: in fact, some prospective studies on dietary intake, lifestyle, and neoplasia identified beta-cryptoxanthin as a protective nutrient (109). In addition, in tissue culture, beta-cryptoxanthin has a direct stimulatory effect on bone formation and an inhibitory effect on bone resorption (110). Epidemiologic studies suggest that the antioxidant potential of dietary carotenoids, such as beta-cryptoxanthin, may protect against the oxidative damage that can result in inflammation. The European Prospective Investigation of Cancer Incidence (EPIC)-Norfolk study, a population-based prospective study of 25,000 subjects, showed that an increase in beta-cryptoxanthin intake, equivalent to one glass of freshly squeezed orange juice every day, was associated with a reduced risk of developing inflammatory disorders such as inflammatory polyarthritis, which is a synovitis affecting two joint groups, and rheumatoid arthritis (111). The Iowa Women's Health Study, a large prospective population-based study of 29,000 women aged 55–69 recently reported a protective effect against the development of RA of a high dietary intake of beta-cryptoxanthin but not of beta-carotene, lutein, and zeaxanthin (112): probably the influence of beta-cryptoxanthin on some markers of inflammatory activity may be stronger than those of other carotenoids. It was recently postulated that this role of circulating antioxidants, as scavengers of FRs and inhibitors of oxidative damage leading to the suppression of inflammation, might also have a role in the prevention of CVD. Further epidemiologic studies displayed that CRP and oxidized LDL-cholesterol concentrations, which have also been linked to the development of CVD, are inversely related to serum concentrations of circulating antioxidants, including beta-cryptoxanthin (113). A recent report found that in the general population obesity is negatively related to serum concentrations of beta-cryptoxanthin and positively related to CRP (114). Thus, we could suppose that beta-cryptoxanthin may also be associated with a reduced cardiovascular risk.

## Beta-carotene

Beta-carotene is one of the most widely studied carotenoids for both its pro-vitamin A activity and its abundance in fruits and vegetables, such as carrot, orange, kale, spinach, turnip greens, apricot, and tomato. It serves as a prehormone that is converted into retinoic acid (RA), which functions as a ligand, regulating the expression of genes involved in metabolic processes (115). Natural beta-carotene comprises several isomers, including all-trans and 9-cis b-carotene. Several epidemiological studies displayed that an abundance of carotenoids in the diet may be protective against many diseases, reducing the risk of CVD and some forms of cancer. In particular, this carotenoid may increase immunological functions by enhancing lymphocyte proliferation and possess antioxidant capacity: the enrichment of LDL with *β*-carotene *in vitro* has been shown to reduce the susceptibility of LDL to oxidative modification (116). Another interesting mechanism to elucidate why carotenoids can prevent CVD is the modulation of vascular NO bioavailability thanks to their reducing activity. In fact, it is well known that one of the earliest pathogenic events in atherosclerosis is represented by the overexpression of cell surface adhesion molecules, which causes the binding of normally non-thrombogenic circulating cells, such as monocytes, to the endothelium: the activation of NF-kB pathway triggers the upregulation of the expression of the vascular cell adhesion molecules (VCAM-1), intercellular cell adhesion molecules (ICAM-1), and E-selectin in response to various inflammatory cytokines (117). NO, constitutively generated by endothelial cells, plays an important role in the maintenance of vascular homeostasis and in the pro-inflammatory response that characterizes the early stages of atherosclerosis: it inhibits the vascular inflammatory response by blocking NF-kB nuclear transfer. A recent study (118) reported that beta-carotene, similar to lycopene, affects NF-kB-dependent expression of adhesion molecule and monocyte– human umbilical vein endothelial cell (HUVEC) interaction induced by TNF-alpha and protect NO bioavailability, thereby reducing TNF-alpha-induced nitro-oxidative stress. In a model of vascular inflammation, the presence of high concentrations of beta-carotene is associated with a significant increase in NO level and bioavailability, as indicated by the increase in cGMP levels: an increased release of NO lead to a downregulation of the expression of NF-kB-dependent adhesion molecules in endothelial cells (119). The maintenance of endothelial NO bioavailability is therefore considered beneficial to endothelial functions and more in general to vascular health. The 9-cis-beta-carotene isomer, present in the highest levels in the alga *Dunaliella bardawil*, showed positive results too: a recent study demonstrated that combined treatment with the drug bezafibrate and Dunaliella powder enhanced the effect of the fibrate on HDL-cholesterol elevation in human apolipoprotein (120). In fact, the effect of fibrates on HDL levels is suggested to be mediated by its binding to peroxisome-proliferator-activated receptor (PPAR) alpha. Upon ligand binding, PPAR-alpha heterodimerizes with the 9-*cis* RA receptor (RXR) and this heterodimer regulates gene expression. The hypothesis is that a combined treatment with fibrate and 9-*cis-beta-*carotene rich powder of the alga *Dunaliella bardawil*, as a source of 9-*cis* RA, would improve the drug's effect on HDL levels (120). Other studies demonstrate that a 9-cis-beta-carotene-rich diet may inhibit atherosclerosis by reducing non-HDL plasma cholesterol concentrations and by inhibiting fatty liver development and inflammation in a mouse model of atherosclerosis (121). Both pathological examination and gene expression showed that a beta-carotene-rich diet reduced inflammation in the livers of mice, by reducing the expression of IL-1a, VCAM-1, and E-selectin. The high-cholesterol diet was shown to induce the expression of several pro-inflammatory genes in the liver and liver inflammation has been suggested to contribute to atherosclerosis; therefore, the reduced levels of these genes in Dunaliella-treated mice can contribute to the protection against diet-induced liver damage and, consequently, atherogenesis. Similar to rexinoids, the 9-cis-rich diet significantly reduced mRNA levels of CYP7a, the rate-limiting enzyme of bile acid synthesis (122) and consequently it may reduce cholesterol absorption in the intestine. The 9-cis-beta-carotene-rich diet also reduced the expression of other genes involved in cholesterol metabolism, ABCG1, ABCG5, and ABCG8. These transporters are expressed in the liver and play a role in excreting cholesterol and therefore, can be expected to reduce atherogenesis. The beneficial effects on plasma lipids in humans suggest that 9-cis-beta-carotene have the potential to inhibit atherosclerosis progression in humans and probably has the potential to reduce the inflammatory process in general. Also, the Manfredonia Study (whose aim was to assess the relationship between asymptomatic carotid atherosclerosis, as defined by carotid intima-media thickness, and inflammatory markers, plasma lipids, and serum antioxidant vitamins) examined 640 subjects with carotid ultrasound investigation, and the collection of medical history and laboratory data, in order to evaluate beta-carotene effects on the cardiovascular system. Among participants with carotid intima-media thickness ≥0.8 mm, body mass index, blood pressure, total cholesterol, LDL-C, triglycerides, uric acid, CRP, and fibrinogen were significantly higher; concentrations of vitamin A, vitamin E, lycopene, and beta-carotene were lower when compared with participants who did not show evidence of carotid atherosclerosis. This study concluded that the optimal control of hypertension, diabetes, and dyslipidemia, in addition to smoking cessation and an adequate intake of antioxidant micronutrients from foods represent a key for the prevention of atherosclerotic disease (123). Finally, beta-carotene resulted implied even in the control of body fat reserves (124): in mature adipocytes, beta-carotene is metabolized to RA, which decreases the expression of PPAR-alpha and CCAAT/enhancer-binding protein, which are key lipogenic transcription factors. Thus, beta-carotene reduces the lipid content of mature adipocytes. Animal studies indicate that diets low in vitamin A favor adipose tissue formation and enhance formation of intramuscular fat. Regulation of fat reserves by dietary vitamin A can be explained by the metabolism of vitamin A to biologically active retinoid derivatives, which then impact the differentiation and function of adipose tissue. The vitamin A derivative all-*trans*-RA has been shown to inhibit adipocyte differentiation in cell culture (125). In mature adipocytes, treatment with pharmacological doses of RA can induce lipolysis, mitochondrial uncoupling, and influence the production of adipokines (126) both in cell culture and mouse models. So, a diet rich in beta-carotene and fat directs toward energy expenditure, but in the absence of beta-carotene, adipocytes store energy as fat. In fact, in humans, circulating beta-carotene levels are inversely correlated with risk of type 2 diabetes and obesity (127), which are important cardiovascular risk factors. However, these benefits are associated with dietary consumption and seem to disappear when beta-carotene is administered as a pharmacological supplement, resulting in harmful effects in some subpopulations: administration of synthetic all-trans b-carotene to smokers seems to increase the incidence of lung cancer and CVD (29). In this respect, the Alpha-Tocopherol, Beta-Carotene Cancer Prevention (ATBC) Study, conducted in Finland as a joint project between the National Institute for Health and Welfare of Finland and the US National Cancer Institute (NCI), deserve a particular mention: this was a randomized, double-blind, placebo-controlled primary prevention trial to determine whether daily supplementation with alpha-tocopherol, beta-carotene, or both would reduce the incidence of lung or other cancers among male smokers. A total of 29,133 men aged between 50 and 69, who smoked at least five cigarettes per day, were recruited and received either alpha-tocopherol (50 mg/day), beta-carotene (20 mg/day) as all-trans-beta-carotene, both supplements, or placebo capsules for 5–8 years until trial closure; researchers reported that men who took beta-carotene had an 18% increased incidence of lung cancers and an 8% increased overall mortality. Vitamin E had no effect on lung cancer incidence or overall mortality. The men taking both supplements had outcomes similar to those taking beta-carotene alone. The adverse effects of beta-carotene appeared stronger in men with a relatively modest alcohol intake (more than 11 g per day; 15 ml of alcohol is equivalent to one drink) and in those smoking at least 20 cigarettes daily (30). The results of both the trial and post-trial follow-up of the ATBC Study, in conjunction with results from the CARET Study (Beta-Carotene and Retinol Efficacy Trial) which compared the effects of beta-carotene plus vitamin A to placebo in 18,314 men and women aged 45–74 who were either smokers or former smokers, evidenced a 28% higher lung cancer incidence and 17% higher overall mortality in the group taking the vitamin supplementation (31); this continues to support the recommendation that beta-carotene supplementation should be avoided by smokers. In this regard, one of the few studies that showed a long-term benefit of supplemental carotenoids deserve a particular mention: the Age-Related Eye Disease Study (AREDS) is a major clinical trial sponsored by the National Eye Institute, which was designed to learn more about the natural history and risk factors of AMD and cataract and to evaluate the effect of high doses of vitamin C, vitamin E, beta-carotene, and zinc in the progression of AMD and cataract. Results from the AREDS showed that high levels of these antioxidants significantly reduce the risk of advanced AMD and its associated vision loss (28). In May 2013, the NEI completed the Age-Related Eye Disease Study 2 (ARDS2), which tested several changes to the formulation by adding omega-3 fatty acids and by substituting lutein and zeaxanthin for beta-carotene, which prior studies had associated with an increased risk of lung cancer in smokers. The study found that while omega-3 fatty acids had no effect on the formulation, lutein and zeaxanthin together appeared to be a safe and effective alternative to beta-carotene. The totality of evidence on beneficial and adverse effects from AREDS2 and other studies suggests that lutein/zeaxanthin could be more appropriate than beta-carotene in the AREDS-type supplements (128). More prolonged follow-up will certainly provide unique and valuable information on the duration of trial effects and potential late effects of intervention with these antioxidant vitamins. Further follow-up will also contribute to our understanding of the biological mechanisms through which such agents affect carcinogenesis and human cancer risk.

## Conclusions

Pathophysiology of many chronic and acute conditions, especially of CVD, is explained by inflammation and oxidative stress. Apart from sex, age, and genetic factors which cannot be modified, lifestyle and dietary intervention can be considered as new important means of prevention and treatment of cardiovascular risk factors. Whilst it would be beneficial not only to practice regular physical exercise, quit smoking, and reduce sodium and cholesterol (106), a higher dietary introduction or supplementation of antioxidant compounds (55), such as polyphenols, vitamins, and carotenoids would also be beneficial. Numerous evidences confirmed that carotenoids possess antioxidant biological properties due to their chemical structure and interaction with biological membranes. In particular, fucoxanthin, astaxanthin, lycopene, and lutein are strong FRs, quenchers of ROS, and NOS, so that their antioxidant and antinflammatory activity may help against cardiovascular risk factors such as markers of inflammation, hyperlipidemia, hypertension, insulin resistance, and obesity. Consequent improvements in blood pressure baseline levels, reduction of inflammation, and correction of dyslipidemias can lead to an improvement of cardiovascular health. Further in-depth efforts in this sense could be studied to define a preventive and therapeutic strategy in order to reduce the risk of developing CVD, with promising applications and no side effects.
